# Construction and Identification of Inflammation-Related TF–mRNA–miRNA Coexpression Network and Immune Infiltration in Parkinson's Disease

**DOI:** 10.1155/padi/2323585

**Published:** 2025-05-07

**Authors:** Zhuzhen Shen, Jieli Zhang, Xiuna Jing, Enxiang Tao

**Affiliations:** ^1^The Eighth Affiliated Hospital of Sun Yat-Sen University Neurology Department, Shenzhen, Guangdong, China; ^2^Department of Neurology, Sun Yat-Sen Memorial Hospital, Guangzhou, Guangdong, China

**Keywords:** bioinformatics analysis, immune infiltration, inflammation-related genes, Parkinson's disease, QPCR, TF–mRNA–miRNA coexpression network

## Abstract

**Background:** Parkinson's disease (PD) is the second most common neurodegenerative disease worldwide. Inflammation, marked by the infiltration of inflammatory mediators and the proliferation of inflammatory cells, is closely linked to PD. This study aims to identify and validate inflammation-related biomarkers in PD and construct a TF–mRNA–miRNA coexpression network through bioinformatics analysis.

**Methods:** The PD-associated dataset GSE7621 and inflammation-related genes were downloaded from the GEO Database and GeneCards platform to obtain inflammation-related differential expression genes (IRDEGs). The key IRDEGs were generated by PPI network analysis. The gene expression levels of the key IRDEGs were validated by blood samples from PD patients using QPCR analysis. We utilized the ENCODE, hTFtarget, CHEA, miRWALK, and miRDB databases to obtain upstream and downstream molecular network models for constructing the TF–mRNA–miRNA interaction network of the key IRDEGs. Finally, based on CIBERSORT algorithm, the associations between IRDEs and immune cell infiltration were investigated.

**Results:** A total of four key IRDEGs (CXCR4, LEP, SLC18A2, and TAC1) were screened and validated. Through biological function analysis, key-related pathways and coexpression networks of PD were identified. These genes may be closely related to the onset of PD. Additionally, we found that increased CD4 T-cell infiltration might be associated with the occurrence of PD.

**Conclusions:** We identified four potential inflammation-related treatment target and constructed a TF–mRNA–miRNA regulatory network. This information provides an initial basis for understanding the complex PD regulatory mechanisms.

## 1. Introduction

Parkinson's disease (PD) is a progressive neurodegenerative disorder characterized by motor symptoms such as rigidity, postural instability, and bradykinesia. These motor deficits are closely associated with the degeneration of dopaminergic neurons in the substantia pars compacta, their projections to the striatum, and the accumulation of α-synuclein aggregates in Lewy bodies within nigral and other neurons [1–3]. PD has become a major global health concern, affecting millions of people worldwide [4]. Despite recent advancements in understanding its mechanisms, the underlying causes of PD remain elusive. PD is now recognized as the fastest-growing neurodegenerative disorder, posing a substantial burden on aging societies [5]. Currently, no disease-modifying therapies are available for PD, and treatment mainly focuses on symptom relief and improving patient's quality of life [6].

Chronic neuroinflammation is believed to play a crucial role in the pathogenesis of PD. The activation of microglia and astrocytes promotes dopaminergic neuron death, which in turn stimulates further inflammation, creating a vicious cycle [7]. Research by Zimmermann et al. has shown increased levels of inflammatory markers in the blood and cerebrospinal fluid of PD patients, with the degree of elevation correlating with the severity of both motor and nonmotor symptoms in PD [8]. In PD, the deposition of proteins such as α-synuclein, Aβ, and tau can elevate many inflammatory factors. For example, overexpression of α-synuclein can drive microglia into a reactive promicroglia modulating the neuroinflammatory process in PD [9, 10]. Multiple studies have shown significant increases in INF-α, IL-1β, and IL-10 levels in the blood and cerebrospinal fluid of PD patients [11, 12]. Therefore, pro-inflammatory cytokines such as IL-1β, IL-6, and TNF-α, as well as anti-inflammatory cytokines such as IL-10, TGF-β, and IL-11, are considered potential biomarkers or therapeutic targets for PD [13]. However, the etiology of inflammation in PD development is still not fully understood and requires further investigation.

Recent studies have highlighted the critical role of miRNA, TF, and mRNA interactions in the pathogenesis of PD. The TF–mRNA–miRNA regulatory network is crucial for gene expression modulation and can significantly influence neuroinflammation processes [14, 15]. miRNAs are known to modulate the expression of genes involved in inflammatory responses by targeting specific mRNAs. For example, miR-124 has been shown to downregulate pro-inflammatory genes by targeting TNF-α mRNA, thereby reducing neuroinflammation [16]. Furthermore, recent evidence indicates that transcription factors such as NF-κB and STAT3 are critical in regulating the expression of pro-inflammatory genes and play a significant role in PD-related neuroinflammation [17].

Previous research has emphasized the importance of immune infiltration in PD. For instance, Cheng Lei et al. found a significant accumulation of M2 macrophages in the brains of PD patients through immune infiltration analysis [18]. Studies have demonstrated that immune cells, including T-cells and macrophages, infiltrate the central nervous system in PD, exacerbating inflammation and neuronal damage [19]. The examination of immune infiltration in PD has revealed that various immune cells, such as microglia, exhibit increased activation and are associated with higher levels of pro-inflammatory cytokines, contributing to the disease's progression [20].

This research utilizes bioinformatics analysis techniques to examine the pathway involved in PD, with a focus on inflammation. Through differential expression analysis, the study identifies inflammation-related genes with significant differences in expression. The relationship between these genes and immune infiltration is further examined through immune infiltration analysis. Additionally, the study explores the potential roles and functions of core genes in regulating inflammation and immune infiltration in PD.

## 2. Materials and Methods

### 2.1. Data Acquisition

We used “Parkinson's disease” and “human beings” as keywords to obtain the gene expression matrix of the GSE7621 dataset [21] from the NCBI Gene Expression Omnibus (GEO) database (https://www.ncbi.nlm.nih.gov/geo/browse/). The GSE7621 dataset includes 16 PD and 9 healthy control (HC) whole substantia nigra samples, which were analyzed on the GPL570 platform. The datasets GSE202666 and GSE8397 were used for validation. Inflammation-related gene sets were obtained from the GeneCards database (https://www.genecards.org/).

### 2.2. Data Preprocessing

Download the Series Matrix File(s) for GSE7621. In R, the probe IDs were mapped to gene symbols using the GPL570-55999 annotation file. When multiple probes corresponded to the same gene, the probe with the highest average expression value was selected to represent the gene's expression. Differential expression analysis was conducted using the “limma” package. The “voom” function with quantile normalization was applied to transform the PD and HC group data into log-CPM values, accounting for mean–variance relationships. Subsequently, a standard linear model was fitted to the normalized data using “lmFit,” followed by contrast fitting with “contrasts.fit.”

### 2.3. Data Processing of DEGs

DEGs between PD and HC samples were identified via the edgeR package with |log_2_ (fold change)| ≥ 1 and *p* value < 0.05. These criteria were selected to balance sensitivity and specificity in our analysis, particularly given our relatively limited sample size. The more stringent fold change threshold (|log_2_ (FC)| ≥ 1) was employed to prioritize genes with substantial biological differences between conditions, thereby reducing potential false positives that might arise from the smaller cohort. The DEGs with log_2_ (fold change) < 0 was considered as downregulated genes, while the GEGs with DEGs with log_2_ (fold change) > 0 was considered as upregulated gene. The results of the DEGs were presented by the heatmaps and volcano plots drawn by gglot2 in R package [22].

### 2.4. Enrichment Analysis (Gene Ontology [GO]/Kyoto Encyclopedia of Genes and Genomes [KEGG])

To explore the potential molecular mechanism of key genes associated with PD and inflammation, we used the Database for Annotation, Visualization, and Integrated Discovery (DAVID) (Version 6.8) (https://david.ncifcrf.gov/home.jsp) [23] to finish GO annotation analysis and KEGG analysis of DEGs. GO analysis includes biological process (BP), cellular component (CC) and molecular function (MF). The significant enriched functions and pathways were selected with *p* value < 0.05 [24]. The results of the enrichment analysis were visualized by using the ggplot2 in R package.

## 3. Construction of the Protein-Protein Interaction (PPI) Network

To obtain the interaction relationship between DEGs, the PPI network was constructed based on all DEGs by the online tool STRING (https://string-db.org/) [25]. Next, we downloaded the interaction information and optimized the PPI network with Cytoscape software (v3.7.0) [26, 27] for better visualization. Based on the PPI network, the top 10 nodes ranked by the degree algorithm were considered as hub genes.

### 3.1. Validation of Core Genes

#### 3.1.1. Patient Selection

Between August 2023 and December 2023, we recruited 10 patients with primary PD at Sun Yat-sen Memorial Hospital, Sun Yat-sen University. The included patients met the clinical diagnosis criteria issued by the United Kingdom Parkinson's Disease Society Brain Bank (UK-PDSBB). The exclusion criteria for recruitment of PD patients were as follows: (1) age < 18 years; (2) limb tremors or bradykinesia due to other causes; (3) presence of cerebrovascular diseases, systemic inflammatory, or autoimmune diseases; (4) presence of malignant tumors. Additionally, 10 age- and sex-matched HCs were recruited from the Physical Examination Center of Sun Yat-sen Memorial Hospital to serve as the control group. All HCs underwent eligibility screening with the same exclusion criteria as the PD patients. This study was conducted in accordance with the Declaration of Helsinki (2013 revision). The study received ethical approval from the Ethics Committee of Sun Yat-sen Memorial Hospital, and all participants provided informed consent (Ethical Review Number: SYSKY-2022-513-01).

#### 3.1.2. Blood Samples

Peripheral blood mononuclear cells (PBMCs) were extracted from 5 mL of peripheral blood using human PBMC separation solution according to the manufacturer's instructions. Subsequently, 1 mL of TRIzol reagent (ThermoFisher) was added to the cell pellet, which was then fully dissolved and stored at −80°C.

#### 3.1.3. RNA Extraction and Quantitative PCR (QPCR)

Total RNA was extracted using the TRIzol method. RNA quantity and quality were measured using a NanoDrop ND-100. The HyperScript™ First-strand cDNA Synthesis Kit was used for reverse transcription. mRNA expression was detected using APExBIO 2X SYBR Green qPCR Master Mix. Relative expression levels were calculated using the 2^−ΔΔCt^ method. The primers used are listed in the Table 1.

#### 3.1.4. Construction of TF–mRNA–miRNA Coexpression Networks

MiRWALK3.0 (https://mirwalk.umm.uni-heidelberg.de/) and miRBD (https://mirdb.org/) are online tools used for predicting downstream target genes. The mRNA–miRNA regulatory network was obtained by overlapping the miRNAs predicted by these two databases. ENCODE (https://www.encodeproject.org/), hTFtarget (https://bioinfo.life.hust.edu.cn/hTFtarget#!/), and CHEA (https://amp.pharm.mssm.edu/ChEA2/) are online tools used for predicting TFs interacting with mRNAs. The TF–mRNA regulatory network was obtained by overlapping the TFs predicted by these three databases. Finally, the TF–mRNA–miRNA regulatory network was constructed and visualized using Cytoscape software.

### 3.2. Immune Infiltration Analysis

The “CIBERSORT” algorithm was applied to calculate the ratios of immune infiltrating cells in PD and HC samples [28]. The number of permutations of default signature matrix was set to 2000, and the standard immune cell expression file was obtained from official website (https://cibersort.stanford.edu/) (Accessed: 7 June 2022). The spearman correlation analysis was performed on candidate genes and infiltrating immune cells, ICGs. The R programming ggplot2 package was used to plot bar graphs to show the distribution of the 22 kinds of immune cells infiltrating in each sample.

### 3.3. Statistics Analysis

The data analysis was conducted using R language software (Version 4.3.1). Statistical significance was determined by considering values with *p* < 0.05.

## 4. Results and Discussion

### 4.1. Identification of DEGs and Inflammation-Related Genes

**Figure 1:**
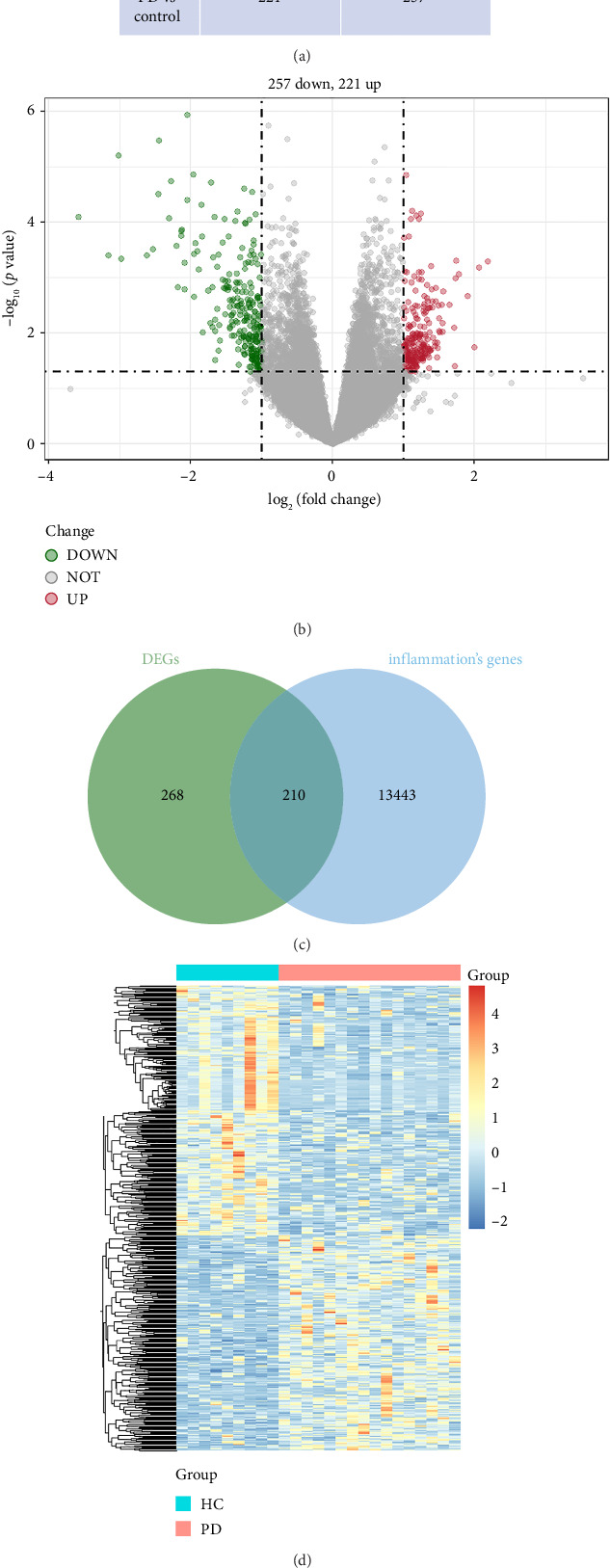
Identification of DEGs and inflammation-related genes. (a) The number of up- and downregulated genes in PD versus HC samples. (b) The volcano plot of DEGs in PD versus HC. (c) Venn diagram presented 210 overlapped IRDEGs. (d) Heatmap of DEGs, in which red means upregulated genes while blue means downregulated genes and grey means nondifferentially expressed genes.

A total of 478 GEDs were obtained from the bioinformatic analysis of PD-related dataset GSE7621 with the criteria of |log_2_FC| ≥ 1 and *p* value < 0.05, in which 221 genes were upregulated and 257 genes were downregulated in PD group (Figure 1(a)). These DEGs were visualized by a heatmap (Figure 1(d)) and a volcano plot (Figure 1(b)). Moreover, 13,653 inflammation-related gene sets were obtained from the GeneCards database, in which 210 genes overlapped with above DEGs and inflammation (Figure 1(c)).

### 4.2. Functional Enrichment Analysis of Inflammation-Related DEGs by GO and KEGG

**Figure 2:**
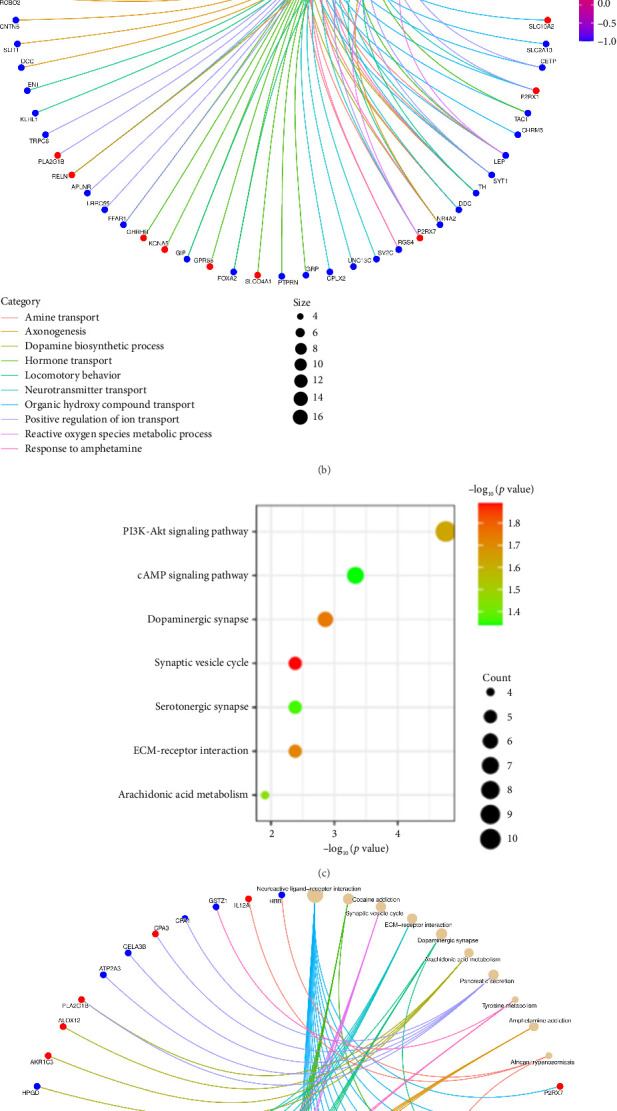
GO and KEGG pathway enrichment results of IRDEGs. (a) The analysis of GO (BP, CC, and MF). (b) IRDEGs involved in the regulation of BP. (c) The analysis of KEGG. (d) IRDEGs involved in the regulation of KEGG pathways.

Functional enrichment analysis was performed on the inflammation-related DEGs by the enriched GO functions and KEGG pathways. GO functional enrichment analysis revealed that the main BP associated with the IRDEGs were dopamine binding, calcium ion binding, growth factor activity, and receptor binding (Figure 2(b)). The CCs where the IRDEGs are located mainly include axon, dendrite, synapse, and secretory granule. The key MFs potentially performed by the IRDEGs are dopamine biosynthetic process, inflammatory response, response to hypoxia, response to lipopolysaccharide, aminergic neurotransmitter loading into synaptic vesicle, dopaminergic neuron differentiation, and positive regulation of reactive oxygen species metabolic process (Figure 2(a)). KEGG pathway enrichment analysis showed that the primary signaling pathways involved in inflammation-mediated PD by the IRDEGs include neuroactive ligand–receptor interaction, dopaminergic synapse, ECM–receptor interaction, PI3K–Akt signaling pathway, and cAMP signaling pathway (Figures 2(c) and 2(d)).

### 4.3. PPI Network and Hub Gene Identification

**Figure 3:**
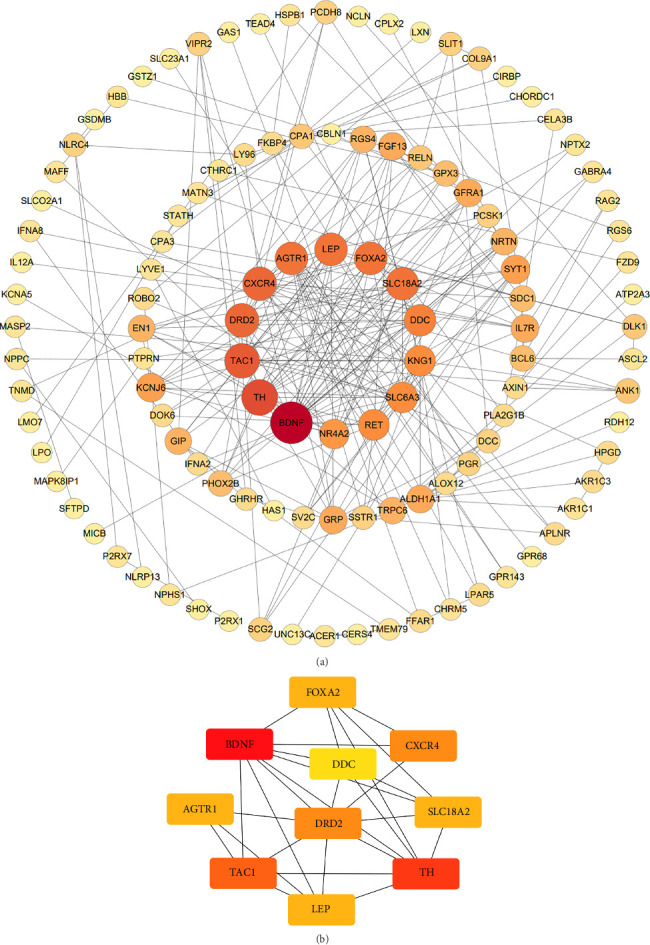
The gene symbol in bold indicates the top 10 IRDEGs. (a) PPI network diagram. The darker the color and the larger the node of the gene in the network, the higher its connectivity with other genes. (b) The top 10 key genes were obtained from the PPI network using degree. The degree value of each gene is represented by the darkness of its color, with darker color indicating a higher degree value.

Based on the STRING online database, a total of 210 IRDEGs were imported into the PPI network complex, which consisted of 114 nodes and 246 edges. All the parameters were set as defaults [29]. The darker the color and the larger the node of the gene in the network, the higher its connectivity with other genes. The cytoHubba plugin was utilized to identify hub genes and select the top 10 genes as key genes using the degree algorithm, as shown in the Figure 3. These top 10 genes, including: AGTR1, BDNF, CXCR4, DDC, DRD2, FOXA2, LEP, SLC18A2, TAC1, and TH, with their relevant information shown in Table 2, are the most important genes in the PPI network and may play a significant role in the pathogenesis of PD.

### 4.4. Validation of Hub Genes Based on QPCR Analysis

**Figure 4:**
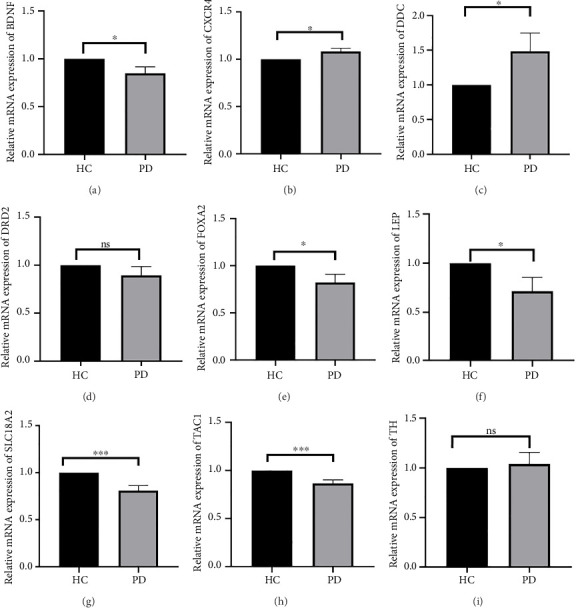
Collection of clinical samples to validate the accuracy and reliability of core genes. ^∗^*p* < 0.05, ^∗∗^*p* < 0.01, ^∗∗∗^*p* < 0.001.

Both the HC and the PD group consisted of 10 patients each. To validate the aforementioned bioinformatics analysis, we employed QPCR analysis. As shown in the Figure 4, the experimental results indicated statistically significant differences in the downregulation of BDNF, FOXA2, LEP, SLC18A2, and TAC1, and the upregulation of CXCR4 in PD patients compared to the control group (*p* < 0.05 or *p* < 0.01 or *p* < 0.001). These findings were consistent with the bioinformatics analysis results. The validation results for DRD2 and TH suggested no statistically significant differences, and thus, they were not classified as the core genes. DDC and AGTR1 were also not classified as core genes due to inconsistencies between experimental validation results and bioinformatics analysis results.

### 4.5. The ROC Curves of Hub Genes Using Another 2 PD Databases From the GEO Database

**Figure 5:**
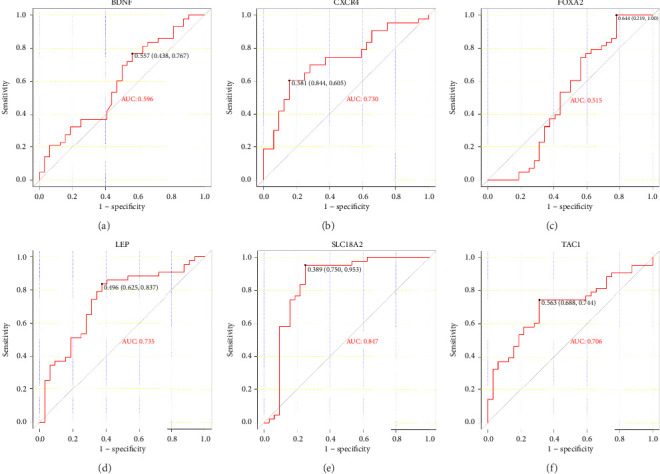
ROC curves of hub genes in GSE8397 and GSE20186.

The ROC curve is used to evaluate the diagnostic performance of hub genes. The area under the curve (AUC) is a measure that combines sensitivity and specificity to describe the intrinsic effectiveness of diagnostic tests. It is generally considered that an AUC greater than 0.7 is statistically significant. As shown in the Figure 5, CXCR4, LEP, SLC18A2, and TAC1 have higher diagnostic value. Therefore, we assume that CXCR4, LEP, SLC18A2, and TAC1 may be the biomarkers related to PD based on our samples.

### 4.6. Construction of TF–mRNA–miRNA Coexpression Networks

**Figure 6:**
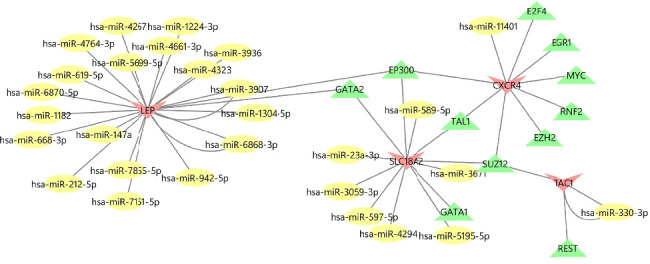
Construction of the TF–mRNA–miRNA coexpression network. Red nodes represent mRNA, green nodes represent TF, and the yellow nodes represent miRNA.

Figure 6 shows the molecular network illustrating the interactions between TFs and miRNA signaling molecules related to the 4 central genes on the ENCODE, hTFtarget, CHEA, MiRWalk3.0, and miRDB online databases. The TF–mRNA–miRNA network comprises 44 nodes and 48 edges. Besides the hub mRNA, EP300, GATA2, TAL1, and SUZ12 are identified as significant TFs.

### 4.7. Validate the Expression of the Coexpression Network Using External Datasets

**Figure 7:**
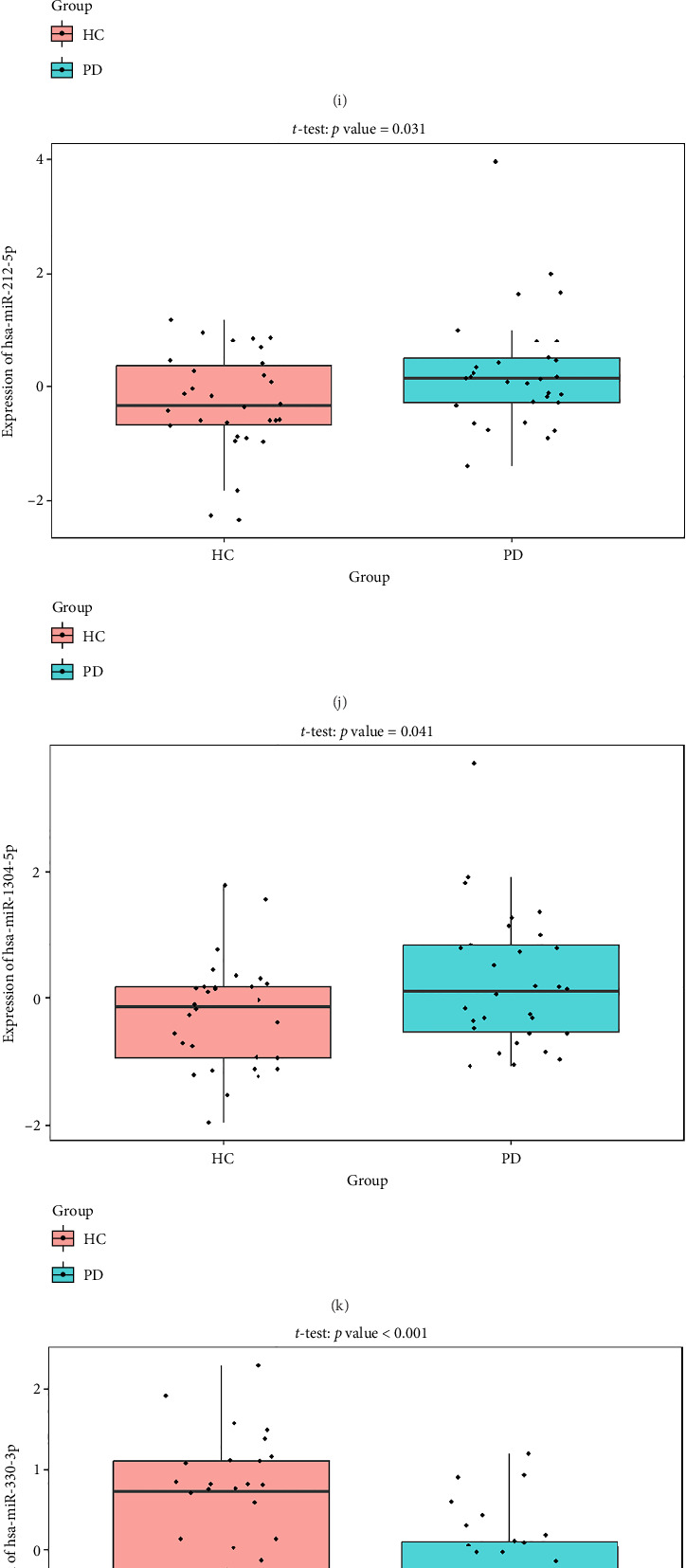
(a–l) The expression of miRNA in GSE202666. (m–p) The expression of mRNA in GSE8397. (q–t) The expression of TF in GSE8397.

The PD-related dataset GSE202666 was downloaded from the GEO database to validate the expression of miRNAs, which includes 30 PD samples and 30 HC samples. The results indicated that the expression of 12 miRNAs—has-miR-212-5P, has-miR-5699-5P, has-miR-3671, has-miR-1304-5P, has-miR-3936, has-miR-1224-3P, has-miR-4267, has-miR-589-5P, has-miR-330-3P, has-miR-4661-3P, has-miR-1764-3P, and has-miR-6870-5P—was statistically significant (Figures 7(a), 7(b), 7(c), 7(d), 7(e), 7(f), 7(g), 7(h), 7(i), 7(j), 7(k), and 7(l)). The expression of mRNA and TFs was validated in the GSE8397 dataset, which includes 29 PD and 18 HC samples. The results showed that the expression trends of CXCR4, SLC18A2, and TAC1 were consistent with those in GSE7621 and exhibited statistically significant differences (Figures 7(m), 7(n), 7(o), and 7(p)). Among the transcription factors, EZH2, GATA1, EP300, and REST showed significant statistical differences (Figures 7(q), 7(r), 7(s), and 7(t)). Based on the TF–mRNA–miRNA coexpression network and validation results, we conclude that GATA1-SLC18A2-has-miR-3671, EP300-SLC18A2-has-miR-3671, EP300-SLC18A2-has-miR-589-5P, GATA1-SLC18A2-has-miR-589-5P, and REST-TAC1-has-miR-330-3P play important roles in the inflammation-mediated onset and progression of PD.

### 4.8. B Cells, CD4 Memory T-Cells, Monocytes, and Neutrophils Were the Most Populations Among Immune Cells by the Immune Infiltration Analysis

**Figure 8:**
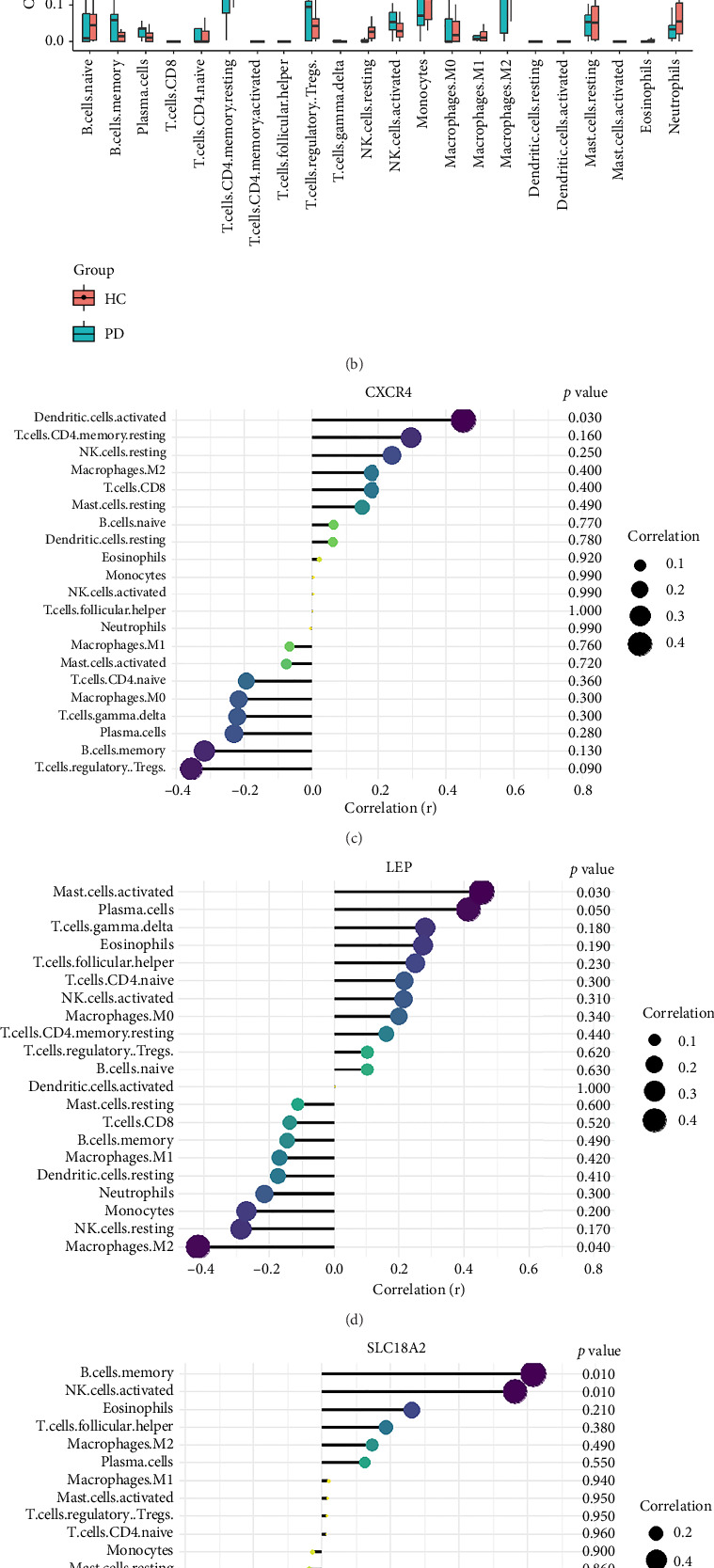
Immune infiltration analysis of GSE7621 and expression of 4 IRDEGs in various immune cells. (a) Correlation analysis between different types of immune cells. (b) Proportions of different immune cells in PD and HC samples. (c–f) Analysis of the correlation between 4 key IRDEGs, including CXCR4, LEP, SLC18A2, and TAC1, and various types of immune cells.

We conducted an immune infiltration analysis using the expression matrix of PD and HC samples in the GSE7621 dataset. The correlation between different species of immune cells and the proportion of different immune cells in all samples were presented in Figures 8(a) and 8(b), respectively. Compared to HC, there is an increased infiltration of B-cells, CD4 memory cells, monocytes, and neutrophils in PD. Furthermore, we conducted additional analysis on the correlation of aforementioned 4 IRDEGs in different immune cells. The results were presented as correlation coefficients and *p* value in the form of a lollipop plot. As shown in the Figures 8(c), 8(d), 8(e), and 8(f), we found that the expression of SLC18A2 and TAC1were positively correlated with the function of B-cells, CD4 memory cells, monocytes, and neutrophils. Additionally, CXCR4 and LEP were positively correlated with the function of B-cells and CD4 memory T-cells, but negatively correlated with function of monocytes and neutrophils.

## 5. Discussion

PD is the second most common neurodegenerative disease, and the gradual loss of dopamine neurons is its main pathological feature. α-synuclein aggregation, oxidative stress, ferroptosis, mitochondrial dysfunction, neuroinflammation, and gut dysbiosis are currently recognized as the pathogenic mechanisms associated with PD. In recent years, there have been fewer studies on the role of inflammation in PD. This article explores the inflammatory biomarkers related to PD through bioinformatics analysis.

CXCR4 is a chemokine receptor protein that plays a pivotal role in modulating various functions within the immune system and neural development. Existing evidence confirms the presence of CXCR4 in dopaminergic neurons of the substantia nigra, the primary region affected in PD [30]. CXCR4 and its ligand CXCL12 regulate neuronal guidance, survival, and inflammatory responses through astrocyte signaling and microglia activation, directly contributing to neurodegenerative progression [31]. In PD pathophysiology, CXCR4 overexpression stimulates microglia to release pro-inflammatory cytokines and neurotoxins, triggering oxidative stress and subsequent apoptosis of dopaminergic neurons. Li et al. demonstrated that blocking CXCR4 signaling with AMD3100 significantly attenuated microglial activation and prevented dopaminergic neurodegeneration in MPTP-induced PD mouse models [32]. This inflammation-mediated neurodegeneration represents a critical mechanistic link between immune dysregulation and dopaminergic system damage in PD.

LEP, a hormone produced by adipocytes, plays a crucial role in regulating basal metabolism through its influence on hypothalamic neuropeptides. In the context of PD, LEP is widely expressed in dopaminergic neurons and possesses dual anti-inflammatory and anti-apoptotic properties [33]. LEP promotes neuroprotection in dopaminergic cells by activating key signaling pathways such as JAK-STAT, MEK/ERK, and GRB2, which inhibit inflammatory responses while enhancing cell survival mechanisms [34]. Dohgu et al. demonstrated that LEP modulates blood–brain barrier permeability, potentially regulating peripheral immune cell infiltration in neuroinflammatory conditions [35]. In experimental models of PD induced by mitochondrial neurotoxins, LEP administration significantly reduced dopaminergic neuron loss by suppressing microglial activation and pro-inflammatory cytokine production. Furthermore, compounds that enhance LEP signaling have been shown to reduce neuronal apoptosis in the substantia nigra in a rat model of PD induced by 6-hydroxydopamine, suggesting therapeutic potential through targeting LEP-mediated inflammatory regulation.

TAC1 is primarily found in the neurons of the nucleus accumbens (NAc), where it encodes preprotachykinin-1, which is then converted into substance P (SP) or neurokinin A. The expression of TAC1 is also observed in dopamine receptor D1-expressing medium spiny neurons, establishing a direct relationship with dopaminergic signaling pathways [36]. Thornton et al. revealed that SP modulates neuroinflammatory responses by regulating microglial activation and cytokine production in neurodegenerative conditions [37]. In PD models, decreased TAC1 expression correlates with dopaminergic neuron loss and increased inflammatory marker expression. Additionally, SP has demonstrated neuroprotective effects by inhibiting microglial activation and reducing proinflammatory cytokine release, potentially mitigating the inflammatory cascade in PD [38]. Although the exact mechanism remains incompletely characterized, the downregulation of TAC1 observed in our study may represent loss of this neuroprotective effect, contributing to exacerbated neuroinflammation and accelerated disease progression.

SLC18A2 is a member of the vesicular monoamine transporter family, responsible for packaging monoamine neurotransmitters including dopamine into synaptic vesicles, thereby playing a critical role in neurotransmission [39]. Beyond this well-established function, recent evidence indicates that SLC18A2 also influences neuroinflammatory processes in PD. Guillot et al. demonstrated that SLC18A2 deficiency leads to cytosolic dopamine accumulation, which increases oxidative stress and activates inflammatory pathways in dopaminergic neurons [40]. This oxidative stress triggers the release of damage-associated molecular patterns (DAMPs), promoting microglial activation and pro-inflammatory cytokine production. Our finding of SLC18A2 dysregulation in PD samples suggests a potential mechanism where impaired vesicular storage capacity leads to dopamine-induced oxidative stress, which subsequently initiates inflammatory cascades. Indeed, Lohr et al. showed that VMAT2-deficient mice exhibit enhanced microglial activation and increased pro-inflammatory cytokine levels in the substantia nigra, preceding dopaminergic neuron loss [41]. This establishes SLC18A2 as a critical link between dopamine homeostasis dysregulation and neuroinflammation in PD pathogenesis.

We validated the TF–mRNA–miRNA co-expression network using an independent dataset and identified several key regulatory axes that may play important roles in inflammation-mediated pathogenesis of PD: GATA1-SLC18A2-hsa-miR-3671, EP300-SLC18A2-hsa-miR-3671, EP300-SLC18A2-hsa-miR-589-5P, GATA1-SLC18A2-hsa-miR-589-5P, and REST-TAC1-hsa-miR-330-3P.GATA1 functions as a transcriptional regulator in the central nervous system and is capable of activating immune responses that lead to neuroinflammation. Previous studies have shown that GATA1 regulates microglial activation by binding to the promoter regions of pro-inflammatory cytokine genes, including IL-1β and TNF-α [42]. In the context of PD, our identification of the GATA1-SLC18A2 axis suggests that GATA1 may contribute to dopamine storage dysfunction through transcriptional repression of SLC18A2, thereby exacerbating oxidative stress and inflammatory responses. This regulatory relationship appears to be modulated by hsa-miR-3671 and hsa-miR-589-5p, both of which are associated with inflammatory pathways. EP300 (p300), a histone acetyltransferase with broad transcriptional coactivator functions, acts as a coactivator of NF-κB (p65) during inflammatory responses [43]. Our identification of the EP300-SLC18A2 regulatory relationships mediated by hsa-miR-3671 and hsa-miR-589-5p indicates the presence of a complex epigenetic mechanism underlying dopaminergic neuroinflammation. Notably, Pan et al. have demonstrated that hsa-miR-589-5p can modulate the activation of the NLRP3 inflammasome, a core component of the neuroinflammatory process in PD [44]. This integrated regulatory network may represent a key mechanism by which inflammatory signaling affects dopamine vesicular transport and subsequent neurotoxicity. The REST-TAC1-hsa-miR-330-3P axis constitutes another important finding from our co-expression network analysis. REST (RE1-silencing transcription factor), also known as NRSF, functions as a transcriptional repressor regulating neuronal gene expression and has been implicated in neuroinflammatory processes. Studies have shown that REST can contribute to inflammation in PD by promoting astrocyte activation [45]. Our identification of the regulatory relationship between REST and TAC1 suggests that REST may control the expression of SP, thereby influencing neuroinflammatory responses in PD. This regulation appears to be fine-tuned by hsa-miR-330-3p.

According to the results of immune infiltration analysis, B-cells, CD4 memory cells, monocytes, and neutrophils are elevated in samples from individuals with PD. This suggests that they may contribute to the progression of PD through inflammation. Abnormal B-cells may contribute to the development and progression of PD by increasing the infiltration of antibodies and cytokines, thereby enhancing neuroinflammation in the central nervous system [19]. The increase in CD4+ memory T-cells is associated with worsening motor dysfunction in PD patients. Several clinical and experimental studies have elucidated the functional impact of CD4+ T-cells in PD pathology. Brochard et al. demonstrated that CD4+ T-cells mediate dopaminergic neuronal cell death in MPTP mouse models of PD, with adoptive transfer experiments showing that CD4+ T-cells, but not CD8+ T-cells, were required for neurodegeneration [19]. Saunders et al. reported that PD patients exhibit increased effector memory CD4+ T-cells with enhanced production of inflammatory cytokines such as IFN-γ and TNF-α in the peripheral blood, which correlates with disease severity [46]. Furthermore, Reynolds et al. found that α-synuclein-specific CD4+ T-cells adopt a Th17 phenotype in PD patients, promoting blood–brain barrier disruption and neuroinflammation [47]. These studies collectively demonstrate that CD4+ T-cells play a critical role in PD pathogenesis through multiple mechanisms, including direct neurotoxicity, cytokine production, and facilitation of neuroinflammation. Monocytes were observed infiltrating the brain in an α-syn-induced mouse model of PD, and this infiltration has detrimental effects. Grozdanov et al. demonstrated that monocytes from PD patients exhibit a proinflammatory phenotype with increased release of cytokines and enhanced phagocytic activity, contributing to neuroinflammation [48]. Neutrophils are the primary source of reactive oxygen species. The increased levels of neutrophils in the peripheral blood of PD patients contribute to the progression of PD by causing elevated oxidative stress levels. Recent work by Earls et al. showed that neutrophil infiltration in the substantia nigra precedes dopaminergic neurodegeneration in PD models, with neutrophil depletion significantly reducing neuronal loss [49].

In the course of our investigation, there are two primary constraints. Firstly, the dataset employed for analysis and verification is of a relatively diminutive scale. Secondly, there exists a dearth of empirical substantiation. Consequently, conducting additional experiments and undertaking clinical validation would be advantageous.

## 6. Conclusions

This study identified four inflammation-related biomarkers (CXCR4, LEP, SLC18A2, and TAC1) and constructed a TF–mRNA–miRNA regulatory network underlying neuroinflammation in PD. We revealed key regulatory axes involving GATA1, EP300, and REST, alongside increased immune cell infiltration. These findings provide novel insights into inflammatory mechanisms in PD pathogenesis and suggest potential therapeutic targets, although further validation with larger cohorts is needed.

## Figures and Tables

**Table 1:** Primer sequences of QPCR.

Gene	Forward primer (5-3)	Reverse primer (5-3)
CXCR4	GCCTGAGTGCTCCAGTAGCC	TGGAGTCATAGTCCCCTGAGC
LEP	TGCCTTCCAGAAACGTGATCC	CTCTGTGGAGTAGCCTGAAGC
SLC18A2	CCTGAATGAAAACGTGCAAGTTG	AGTAGTCCTATGAAAGGGTTGGT
TAC1	GTTATGGGCATCGACGAGTT	AGACCCACGTGACATTCTCC
GADPH	ACAACTTTGGTATCGTGGAAGG	GCCATCACGCCACAGTTTC

**Table 2:** 10 hub genes identified by degree algorithms of cytoHubba.

Gen symbol	Description	Log_2_FC	*p* value	Regulation
AGTR1	Angiotensin II receptor type 1	−2.131	0.0001458	Down
BDNF	Brain-derived neurotrophic factor	−1.063	0.0256349	Down
CXCR4	C-X-C motif chemokine receptor 4	1.156	0.0110275	Up
DDC	Dopa decarboxylase	−3.166	0.0003932	Down
DRD2	Dopamine receptor D2	−1.166	0.0003956	Down
FOXA2	Fork head box A2	−1.597	0.0071985	Down
LEP	Leptin	−1.008	0.0483689	Down
SLC18A2	Solute carrier family 18 member A2	−3.02	0.0000079	Down
TAC1	Tachykinin precursor 1	−1.26	0.0113367	Down
TH	Tyrosine hydroxylase	−3.58	0.0000788	Down

## Data Availability

The data that support the findings of this study are available in the GEO database at (https://www.ncbi.nlm.nih.gov/geo/query/acc.cgi?acc=GSE7621), reference number GSE7621. These data were derived from the following resources available in the public domain: GEO database (https://www.ncbi.nlm.nih.gov/geo/).
